# Urban environmental and climatic correlates of pediatric respiratory infection: microscale spatiotemporal evidence from Fuzhou, China

**DOI:** 10.1186/s12887-026-06993-2

**Published:** 2026-05-30

**Authors:** Yangbin Dong, Yanying Rao, Jie Zhang, Qiuyu Tang, Zhanyong Wang

**Affiliations:** 1 College of Transportation and Civil Engineering, Fujian Agriculture and Forestry University, Fuzhou, 350108 China; 2 Department of Radiology, Fujian Children’s Hospital (Fujian Branch of Shanghai Children’s Medical Center), College of Clinical Medicine for Obstetrics & Gynecology and Pediatrics, Fujian Medical University, Fuzhou, Fujian 350014 China; 3 Pediatric Intensive Care Unit‌, Fujian Children’s Hospital (Fujian Branch of Shanghai Children’s Medical Center), College of Clinical Medicine for Obstetrics & Gynecology and Pediatrics, Fujian Medical University, Fuzhou, Fujian 350014 China

**Keywords:** Mycoplasma pneumoniae pneumonia, Spatial epidemiology, Urban environment, Urban subdistrict, Machine learning

## Abstract

Urban environmental and climatic conditions are closely linked to pediatric respiratory health, yet their associations with specific infectious diseases such as mycoplasma pneumoniae pneumonia (MPP) remain poorly understood. To address this gap, this study investigated the spatiotemporal dynamics of pediatric MPP across 46 urban subdistricts in Fuzhou, China. Using global and local spatial autocorrelation, space–time scan statistics, and interpretable machine learning models, we assessed both incidence patterns and progression to severe disease, as well as their built-environment correlates. Key findings include: (1) A pronounced seasonality of MPP is observed, with autumn–winter peaks and summer troughs. High-risk clusters are concentrated in central urban areas, whereas peripheral regions exhibit weaker clustering. These patterns are consistent with variations in climate, population density, transport networks, greenspace, and school-term cycles. (2) The autumn–winter period represents the critical risk window, encompassing a large share of cases and repeatedly centering on repeatedly high-incidence subdistricts. Demographically, boys and children aged 5–9 years face the highest risk, whereas the 10–14 age group—particularly girls—shows the lowest incidence. (3) Machine learning identified age, near-road exposure, and selected land-use/amenity intensities as core predictors, especially for severe MPP cases. Age exhibits a non-monotonic incidence profile, while distance from primary roads follows a steep inverse gradient, with elevated risk confined to a 100–150 m roadside buffer zone. School density shows a monotonic positive association, whereas greenspace is a broadly negative association. These findings may help inform seasonally tailored, microscale interventions, strengthened school health monitoring, and targeted urban design in high-prevalence corridors.

**Supplementary Information:**

The online version contains supplementary material available at 10.1186/s12887-026-06993-2.

## Introduction

Community-acquired pneumonia (CAP) is a common reason for pediatric clinic and emergency visits and for hospitalization, and Mycoplasma pneumoniae is one of the main causes of CAP in children [1]. In many places, Mycoplasma pneumoniae pneumonia (MPP) makes up a large share of pediatric CAP (often ~ 10%–40%), and outbreaks often follow multi-year cycles, with a clear peak about every 3–7 years. MPP also shows a strong age pattern. Most cases occur in preschool and school-aged children, which fits with close contact in childcare centers and schools. In addition, most infections are mild and improve on their own. Still, some children develop severe or refractory MPP, with persistent high fever, lung consolidation, hypoxemia or respiratory failure, and sometimes extrapulmonary complications [2, 3]. These severe cases often last longer and use more inpatient and intensive-care resources [4–7].

MPP patterns typically change over time within a year and across years. Many studies report both seasonal peaks and longer cycles. Peaks are often seen in autumn and winter, but timing can differ by climate and local behavior [7]. The COVID-19 pandemic temporarily disrupted these established patterns. Non-pharmaceutical interventions (NPIs) reduced many respiratory infections, including MPP [8]. Following the relaxation of NPIs, many regions experienced a rapid rebound, and some reported resurgence waves [9]. These changes make it important to re-check when high-incidence periods occur and whether severe cases rise at the same time.

MPP also exhibits marked spatial heterogeneity, even within a single city. Outbreaks often happen in semi-enclosed or high-contact settings, with clusters reported in school and training institutions, highlighting that dense contact networks can drive spread [10, 11]. However, much of the existing evidence relies on aggregated data at national, regional, or city levels [4, 12]. Such coarse resolution may hide fine-grained differences between neighborhoods and make local outbreak centers hard to detect. This spatial mismatch is particularly consequential, because prevention messages and surge planning are typically designed and implemented at district, subdistrict, or community levels. Several factors may work together to shape MPP risk. For example, time-series studies link changes in temperature and humidity with MPP incidence [5, 13, 14]. Air pollution such as traffic-related particulate matter pollution also can increase respiratory vulnerability and worsen clinical course [15, 16]. The built environments including near-road settings, land-use intensity, travel corridors, greenspace, and the placement of schools and services further affect both exposure and contact. However, many studies still test one driver at a time, and they often use large spatial units, so multi‑factor coupling effects are not well measured [17–20].

A variety of methods have been used to analyze the spatiotemporal dynamics of MPP, but they have limits to varying degrees. For instance, retrospective cohorts and regression models only can describe broad patterns of MPP [13, 21], and general spatial clustering methods can find coarse hotspots [22, 23]. Still, these approaches often do not capture changing space–time patterns at local scales [22]. Space–time scan statistics, such as SaTScan, can detect clusters in both space and time, and also can point to likely outbreak centers, while they are less used for MPP analysis, especially at local geographic units [7, 12]. Coupled with interpretable machine learning with SHAP analysis, the fine-scale spatiotemporal variations of MPP and the contributions of multiple influencing factors can be fully understood.

To address these gaps, we integrated global/local spatial autocorrelation, space–time scan statistics, and a SHAP-based interpretable machine-learning framework to analyze confirmed pediatric MPP cases across 46 subdistricts in central urban area in Fuzhou, China (June 2023–May 2024). Our objectives are: (1) to delineate fine-scale spatiotemporal patterns to identify high-incidence neighborhoods and critical outbreak windows; (2) to quantify urban environmental correlates of both incidence and progression to severe disease—with emphasis on transportation infrastructure (arterial-road proximity/density, parking facilities) and land-use patterns (industrial/catering zones) alongside greenspace; and (3) to discuss plausible pathway linking these exposures to pediatric respiratory risk. By making neighborhood-level patterns explicit, this study provides spatial evidence to support precision prevention and targeted resource allocation in high-risk urban corridors.

## Data and methods

### Study area and data collection

**Fig. 1:**
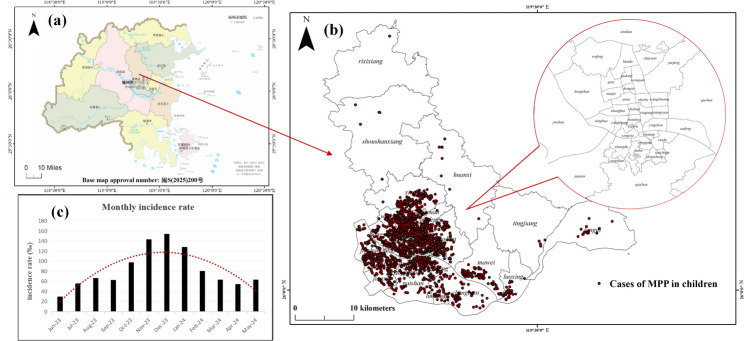
Study area (**a**), spatial distribution (**b**) and monthly average incidence rate (**c**) of pediatric MPP in the study area

The scope of this retrospective, hospital-based study includes the five main districts of Fuzhou City in China, namely Gulou District, Taijiang District, Cangshan District, Jin'an District, and Mawei District, as shown in Fig. 1. The five main districts consist of 46 subdistricts covering a total area of 1,035.73 square kilometers and an estimated population of approximately 3.3042 million, based on the 2023 census data. Among these, the population of children aged 0–14 is approximately 527,100. The study region exhibits a high population density with a significant concentration of children, rendering it particularly valuable for public health research.

The study data were obtained from four primary sources. First, pediatric pneumonia case data from the five districts of Fuzhou City were provided by the Fujian Provincial Children’s Hospital, covering 3,757 diagnosed pediatric MPP cases from June 1, 2023, to May 31, 2024. For each case, information on the onset date, age, gender, residential address, and diagnostic details was recorded. Second, subdistrict zoning data were sourced from the Fuzhou City Bureau of Natural Resources and Planning, including administrative boundaries and geographic information for the 46 subdistricts across the five districts. For ease of analysis, these subdistricts were sequentially numbered, as presented in Table 1. Third, pediatric population data were obtained from the Seventh National Census, as published by both the National Bureau of Statistics and the Fuzhou City Bureau of Statistics, and are used to calculate the incidence rate of pediatric MPP in each subdistrict (see Eq. (1)). Fourth, urban environmental data obtained from Fuzhou Geographic Information Public Service Platform included: 1) built environment elements (building footprints, road network configurations, land cover); 2) spatial distributions of points of interest (POI) across eight categories (transportation hubs, commercial facilities, industrial zones, cultural/educational institutions, medical facilities, etc.).

1 $$\mathrm{IRPMPP}=\left(\frac{\mathrm{NPCSM}}{\mathrm{TPPS}}\right)\times 1000\boldsymbol{\permil }$$

where IRPMPP is the incidence rates of pediatric MPP, NPCSM is the number of pediatric MPP cases in a subdistrict in a month, TPPS is the total pediatric population in the subdistrict. Using the census data, the incidence rate of pediatric MPP in each subdistrict was calculated. It was found that, except for a significant increase in incidence rate from February to March 2024 (which corresponds to the school start period), the incidence rate throughout the year exhibited distinct seasonal variations, as shown in (Fig. 1(c)). Therefore, the study period was divided into four seasons: summer (June 2023-August 2023), autumn (September 2023-November 2023), winter (December 2023-February 2024), and spring (March 2024-May 2024).

**Table 1:** Serial numbers of the 46 subdistrict units across the five districts within the study area

SN	Subdistrict	SN	Subdistrict	SN	Subdistrict	SN	Subdistrict
1	Antai	13	Gudong	25	Luozhou	37	Wenquan
2	Aofeng	14	Gushan	26	Mawei	38	Wufeng
3	Cangqian	15	Guxi	27	Nanjie	39	Xiadu
4	Cangshan	16	Hongshan	28	Ninghua	40	Xiangyuan
5	Cangxia	17	Houjie	29	Rixi	41	Xindian
6	Chating	18	Huada	30	Sanchajie	42	Xingang
7	Chayuan	19	Huanxi	31	Shangdu	43	Yangzhong
8	Chengmen	20	Jianxin	32	Shanghai	44	Yizhou
9	Dongjie	21	Jinshan	33	Shoushan	45	Yingzhou
10	Dongsheng	22	Langqi	34	Shuibu	46	Yuefeng
11	Duihu	23	Linjiang	35	Tingjiang		
12	Gaishan	24	Luoxing	36	Wangzhuang		

Given the uneven spatial distribution of medical resources, diagnoses and reporting are more likely to occur where medical facilities are concentrated; therefore, consequently, spatial differences in incidence may partly reflect disparities in access to care. To reduce this bias, we adopted the following measures. (1) We narrowed the study area from the entire municipality of Fuzhou to the hospital’s primary service area—the five urban districts, which contributed ≥ 65% of pediatric visits during the study period. Travel distances to the hospital within this area were short (median 7.45 km, IQR 6.94–8.56 km), indicating an accessibility-saturated context. (2) Cases were geocoded to residential addresses and assigned to the subdistricts where patients resided. (3) We conducted descriptive accessibility checks by plotting distance vs. number of cases and distance vs. population-standardized incidence (per 1,000 population) (Figs. S1–S2), and we reported the Spearman rank correlation between distance and incidence (ρ = − 0.36, two-sided *p* < 0.001) as well as between distance and case counts (ρ = − 0.067, *p* = 0.746). These checks were used solely to assess whether a monotonic distance-decay pattern consistent with systematic under-ascertainment was present, and were not used in model estimation. (4) After analyzing the pooled dataset for the five districts, we conducted district-stratified analyses to verify that the main spatial patterns were not driven by any single district.

This retrospective, hospital-based observational study was approved by the Ethics Committee of Fujian Children’s Hospital (Fujian Branch of Shanghai Children’s Medical Center) (approval No. 2025ETKLRK04006). The study used routinely collected electronic medical records of hospitalized children with Mycoplasma pneumoniae pneumonia (MPP) in the five urban districts of Fuzhou between 1 June 2023 and 31 May 2024 for secondary analysis. All data were de-identified before analysis. The Ethics Committee of Fujian Children’s Hospital (Fujian Branch of Shanghai Children’s Medical Center) not only approved the study, but also waived the requirement for written informed consent to participate from parents or legal guardians.

### Research methods

#### Spatial autocorrelation analysis

We used the global Moran’s I to test spatial autocorrelation in pediatric MPP incidence at the subdistrict level across the five districts of Fuzhou City. Moran’s I ranges from − 1 to 1. Values in (0, 1] mean positive spatial clustering, and larger values mean stronger clustering. Values in [24]. Local results were grouped into four patterns: high–high, high–low, low–high, and low–low [25, 26].

#### Spatiotemporal scanning analysis

Spatiotemporal scan analysis is a statistical method used to find disease clusters across space and time. It tests whether case counts group together in certain places and periods more than expected by chance. This approach has been widely adopted in epidemiological studies to identify localized outbreaks, assess spatiotemporal risk patterns, and inform targeted public health interventions [27]. The theoretical foundation of this method lies in the space–time scan statistic which employs a dynamic cylindrical window to scan the study area over predefined temporal interval. The spatial dimension of the cylinder represents candidate geographical regions, while its height corresponds to time periods. Within each window, the observed number of cases is statistically compared against the expected number under the null hypothesis of random distribution, typically modeled using Poisson or Bernoulli probability frameworks.

We used a Poisson model to account for differences in population size across subdistricts and to improve cluster detection. For the space–time scan, we first tested the effect of the temporal window by running a sensitivity analysis with the maximum window set to 1–5 months, and then compared primary-cluster membership across settings using the Jaccard index. Specifically, we summarized the primary-cluster time frame, relative risk (RR), log-likelihood ratio (LLR), and number of subdistricts for each candidate window, and quantified cluster-membership overlap relative to the 3-month setting using the Jaccard similarity coefficient. We then selected the window that showed stable cluster patterns and fit the seasonal changes in MPP incidence. Because the observed main epidemic phase in our data extended from November 2023 to January 2024, and because pediatric MPP in our study area showed a clear autumn–winter seasonal peak, the 3-month setting was considered most appropriate for capturing the core seasonal epidemic period while retaining acceptable primary-cluster stability and temporal interpretability. In general, the 3-month maximum temporal window was selected as the main specification, and the remaining settings were treated as sensitivity analyses. Furthermore, we limited the maximum spatial window to 50% of the total population to avoid overly large clusters while still capturing local high-risk areas. Finally, we assessed statistical significance using 999 Monte Carlo simulations with a threshold of *p* < 0.05 to reduce false positives.

To assess the stability of primary-cluster membership under varying parameter configurations, we calculated the Jaccard similarity coefficient (J) between the sets of street units assigned to the primary cluster under two distinct settings, denoted A and B.

2 $$J(A,B)=\frac{\left|A\cap B\right|}{\left|A\cup B\right|}$$

where $\left|.\right|$ denotes set cardinality. J ranges from 0 to 1, with J = 1 indicating identical membership and J = 0 indicating no overlap. Following common practice, values J ≥ 0.80 were interpreted as high stability.

The intensity of identified clusters was also quantified using two key metrics. One is Relative Risk (RR), which is defined as the ratio of observed to expected cases within the scanning window relative to the background area, calculated as:

3 $$RR=\frac{\frac{{C}_{in}}{{E}_{in}}}{\frac{{C}_{out}}{{E}_{out}}}$$

where *C*_*in*_ and *C*_*out*_ denote observed cases inside and outside the window, respectively, while *E*_*in*_ and *E*_*out*_ represent expected cases under spatial randomness.

Another metric is Log–Likelihood Ratio (LLR), a statistical significance measure for cluster detection, expressed as:

4 $$LLR=2\left({C}_{in}ln\left(\frac{{C}_{in}}{{E}_{in}}\right)+{C}_{out}ln\left(\frac{{C}_{out}}{{E}_{out}}\right)\right)$$

Specifically, when RR > 1, it indicates that the risk of cases within the window is higher than in the background area, and when RR < 1, it indicates that the risk of cases within the window is lower than in the background area. When *C*_*in*_ > *E*_*in*_, the LLR is positive, indicating that the number of cases within the window is significantly higher than expected, suggesting a potential cluster. When *C*_*in*_ < *E*_*in*_, the *LLR* is negative, indicating that the number of cases within the window is lower than expected, with no abnormal clustering. The LLR value is used to select the most significant clustering window, where the window with the highest LLR value is considered the most significant cluster. When *p* < 0.05, a larger LLR value indicates a higher likelihood of spatiotemporal clustering in the region within the window. The statistic LLR, constructed using the theoretical and observed incidence rate numbers, describes the degree of clustering. The region with the highest LLR is classified as a Type I cluster, while others are classified as Type II clusters.

#### Interpretable machine learning for pediatric MPP onset and severity determinants

In order to investigate the dual objectives of elucidating associations between urban built environment factors and pediatric MPP incidence, and to identify critical built environment determinants influencing progression to severe pneumonia among confirmed MPP cases, this study first established a spatial database using ArcGIS 10.8 and implemented a two–tiered spatial analytical framework to investigate environmental determinants: 1) Proximity analysis was conducted to compute minimum Euclidean distances between high–risk case locations and hierarchical road network elements (expressways, arterial roads, secondary trunk roads); 2) A 15–minute walkable community model was developed following healthy city principles by constructing 1–km radial buffers around case locations. Because adjacent buffers could overlap, simultaneous extraction might have led to duplicated counting or biased summaries of built-environmental features. Therefore, the iterative modeling function in ArcGIS was used to process each buffer separately rather than extracting all buffers simultaneously. Spatial parameters including floor area ratio (FAR), land cover density, and points of interest (POI) were then extracted from the original spatial layers and quantified for each case-specific buffer.

We developed and evaluated two prediction tasks—(i) pediatric MPP incidence onset and (ii) progression to severe disease—using the same preprocessing and modeling pipeline while training and assessing each task independently. Numeric predictors were median imputed. To mitigate multicollinearity, for any pair with Spearman ∣ρ∣ ≥ 0.90 we removed one variable (retaining the higher-variance feature), and reported variance inflation factors; z-standardization was applied only for the L1-regularized logistic model. Commonly-used four machine learning classifiers including Naïve Bayes (NB), L1-regularized logistic regression (L1-Logit), Random Forest (RF), and XGBoost were selected and estimated in this study [28, 29]. For each endpoint, class imbalance was addressed using class-balanced learning objectives where applicable during model fitting. Performance was estimated via stratified five-fold cross-validation with three-fold inner tuning for L1-Logit, RF, and XGBoost using prespecified candidate settings and mean ROC–AUC as the selection criterion, whereas NB was fitted using its default specification, followed by post-hoc probability calibration using Platt’s sigmoid on training data only. All evaluations were confined to held-out folds to prevent information leakage. The detailed tuning and calibration scheme for e-ach classifier is summarized in Supplementary Table S3. The evaluation framework comprised discrimination (ROC–AUC, PR–AUC), probabilistic accuracy (Brier score), and calibration (intercept, slope, and calibration plots), plus operating characteristics at a prespecified 80% sensitivity (reporting the corresponding threshold, specificity, PPV, and NPV); clinical utility was quantified by decision-curve analysis (net benefit across thresholds). For both tasks, models were ranked primarily by higher AUC and lower Brier, with calibration and DCA as secondary criteria; the top model was then refit on the full analysis dataset. Interpretability was provided using TreeSHAP for the selected model, summarizing global importance via mean absolute SHAP values and visualizing effect shapes with dependence and violin plots whose point colors encode relative feature values; these explanations characterize model contributions rather than causal effects. All analyses were conducted in Python with fixed random seeds to ensure reproducibility.

## Results

### Overall temporal and spatial characteristics of pediatric MPP incidence

**Fig. 2:**
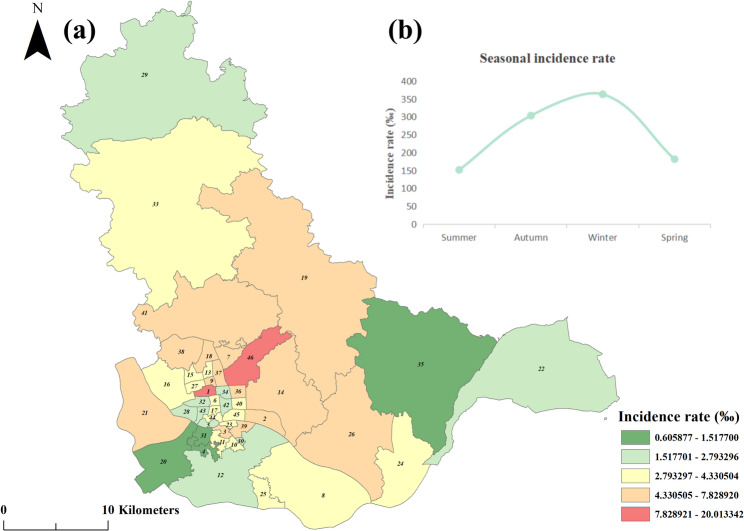
Spatial distribution of subdistrict-level pediatric MPP incidence (**a**) and seasonal variation of pediatric MPP incidence (**b**) within the study area

Spatial analysis of pediatric MPP annual incidence (Fig. 2a) reveals significant spatial heterogeneity at the subdistrict level. High–incidence zones (7.83–20.01 cases per 1,000) are predominantly clustered in Subdistricts 1 and 46, while moderate–incidence areas (2.79–7.83 cases per 1,000) form concentric patterns concentrated in the central urban belt. Low–incidence regions (0–2.79 cases per 1,000) exhibit a dispersed spatial distribution across both central and peripheral zones. Fig. 2b further delineates the characteristic seasonal fluctuations, with incidence rates peaking in winter (350–380 cases per 1,000), followed by autumn (280–300 cases per 1,000), while significantly lower rates are observed in summer and spring (150–200 cases per 1,000).

**Fig. 3:**
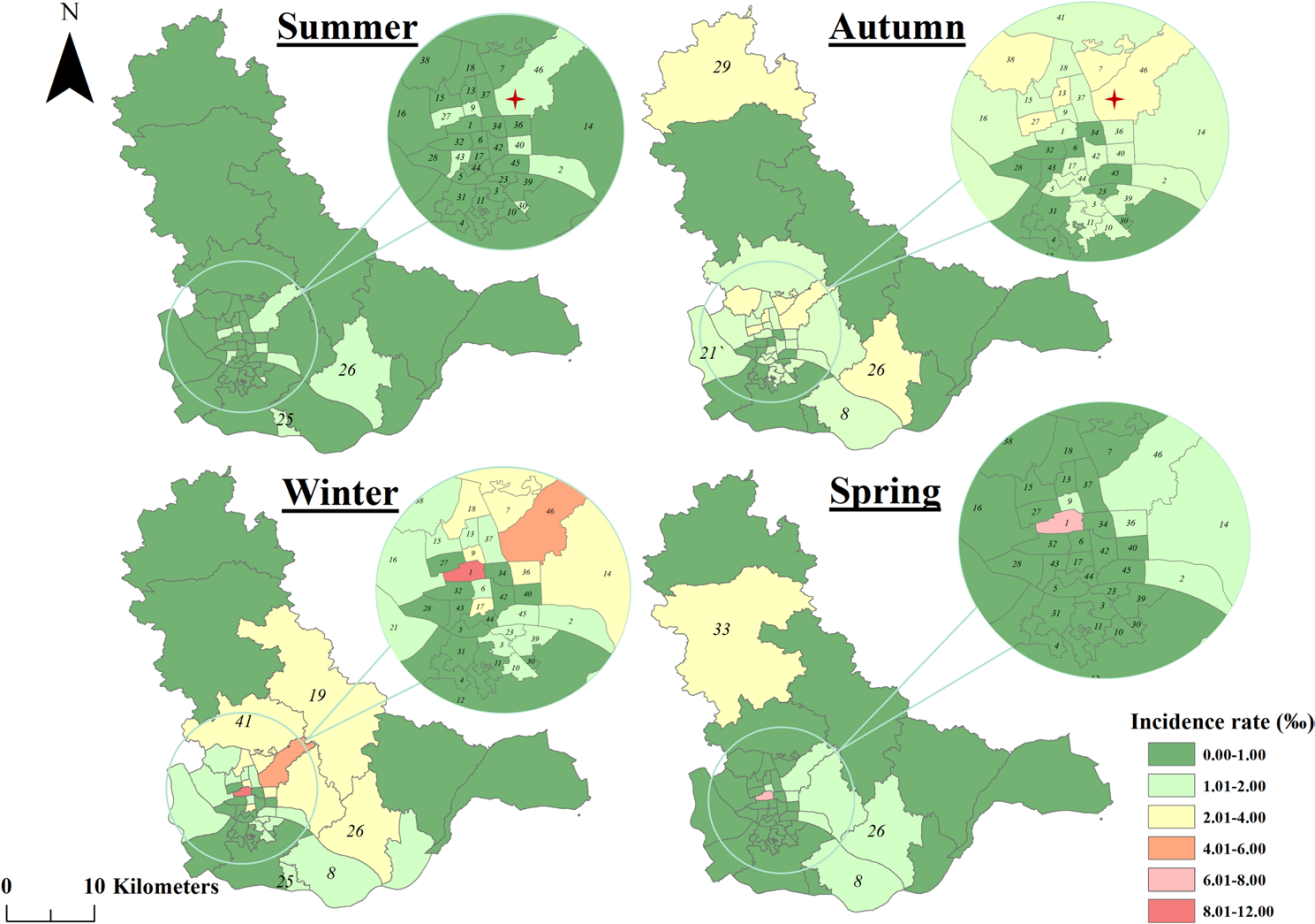
Spatial distribution of subdistrict–level pediatric MPP incidence across four seasons. Note: Red asterisks denote subdistricts with the highest seasonal incidence, consistently marking peak morbidity areas across temporal analyses

Spatial patterns of pediatric MPP incidence across the four seasons demonstrate distinct epidemiological gradients (Fig. 3). Summer exhibits lowest prevalence (0–2 cases per 1,000) with limited spatial clustering. Autumn witnesses moderate escalation (1–4 cases per 1,000), showing centrifugal diffusion from central urban zones to southeastern and northwestern peripheries, though peak incidence loci remains consistent with summer patterns. Winter manifests epidemic intensification, achieving annual maxima (2–12 cases per 1,000) with confluent high–risk corridors spanning northwestern and southeastern sectors. The red asterisk–marked core zone expands centrally (8–12 cases per 1,000), demonstrating 3.8–fold higher incidence than adjacent subdistricts. Spring displays risk mitigation with contracted high–incidence boundaries, yet the asterisk–designated areas maintain 87.6% spatial overlap with winter's epicenters. Notably, Subdistricts 1 and 46 persistently exhibit asterisk–marked hyperendemicity throughout all seasonal cycles.

### Spatiotemporal clustering characteristics of pediatric MPP incidence

#### Spatial autocorrelation patterns of pediatric MPP incidence

The global Moran's I statistics shown in Table 2 are positive and statistically significant across all four seasons, indicating that pediatric MPP incidence at the subdistrict level exhibits global spatial autocorrelation rather than a random spatial pattern. Under the inverse-distance specification, the Moran’s I values are modest, ranging from 0.0553 to 0.0677, with the highest values observed in autumn and winter (0.0677 and 0.0629, respectively), suggesting relatively stronger—though still modest—global clustering during these seasons. Robustness analyses using alternative spatial weights matrices yielded consistently positive and statistically significant Moran’s I values across all seasons, with stronger clustering under contiguity-based specifications than under inverse-distance weighting. These findings indicate that the presence of global spatial autocorrelation is robust in direction and significance, although its magnitude depends on the definition of neighborhood structure.

**Table 2:** Global spatial autocorrelations of subdistrict–level pediatric MPP incidence across four seasons

Season	Weight matrix	Moran I	*p*-value	z-value
Summer	Inverse distance	0.0584	0.0015	3.1747
Summer	Rook contiguity	0.1827	0.0126	2.4763
Summer	Queen contiguity	0.1618	0.0231	2.2714
Fall	Inverse distance	0.0677	0.0004	3.5384
Fall	Rook contiguity	0.2539	0.0008	3.3392
Fall	Queen contiguity	0.2351	0.0021	3.1534
Winter	Inverse distance	0.0629	0.0007	3.3576
Winter	Rook contiguity	0.2442	0.0011	3.2549
Winter	Queen contiguity	0.2255	0.0021	3.0723
Spring	Inverse distance	0.0553	0.0021	3.0654
Spring	Rook contiguity	0.2946	< 0.0001	3.9550
Spring	Queen contiguity	0.2803	0.0001	3.8339

**Fig. 4:**
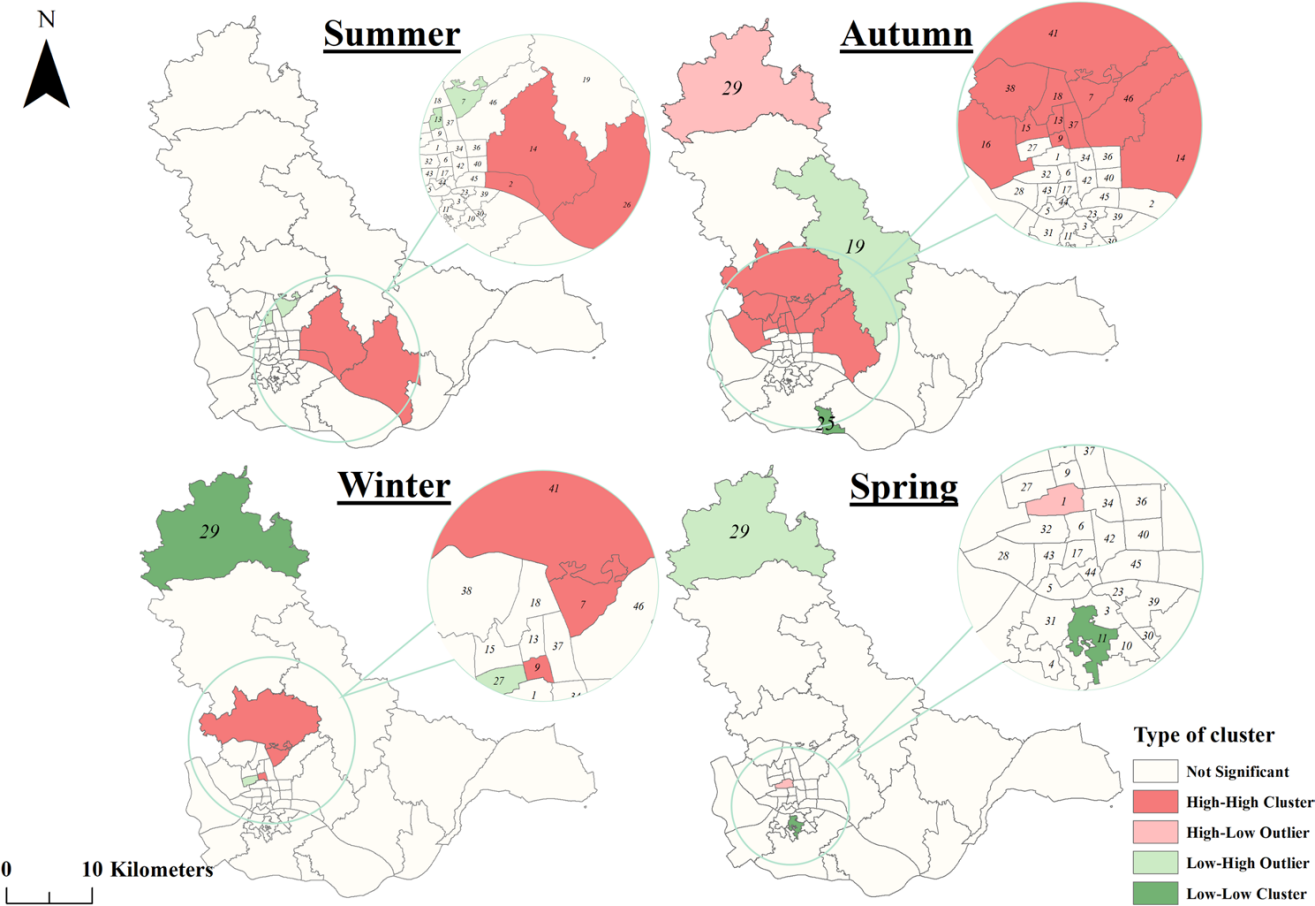
Local spatial autocorrelation of subdistrict–level pediatric MPP incidence across four seasons

Local spatial autocorrelation analysis reveals spatiotemporal clustering patterns of pediatric MPP incidence across subdistricts in four seasonal cycles (Fig. 4). Summer shows weak spatial dependence, with insignificant clusters covering > 60% of the area. Sporadic High–High clusters emerge in southeastern Subdistricts 2,14,26, indicating localized hotspots. Isolated Low–High clusters in central urban zones suggest cold spots surrounded by high–risk peripheries. Autumn exhibits intensified heterogeneity, with expanding High–High clusters (25%–30%) across central Subdistricts 7, 9, 13–16, 18, 37, 38, 41, and 46, overlapping densely populated urban cores. Winter demonstrates epidemic contraction, with High–High clusters retreating to Subdistricts 9, 41, and 46, and southeastern hotspots diminishing substantially. Emerging High–Low clusters suggest dynamic shifts in inter–zonal transmission risks. Spring displays predominantly random patterns (> 80% insignificant areas), with scattered High–Low and Low–Low clusters in central regions. Crucially, autumn–winter High–High clusters show 92.3% spatial concordance with annual peak incidence periods and asterisk–marked hyperendemic foci.

#### Spatiotemporal aggregation patterns of pediatric MPP incidence

The specific subdistrict membership of the primary and secondary clusters under each temporal-window setting is provided in Supplementary Table S1, allowing direct comparison across the main and sensitivity analyses. Sensitivity analyses across 1–5-month maximum temporal windows show that the primary high-risk clusters remain broadly stable across settings (Table S2). The 1-month, 4-month, and 5-month settings each show high overlap with the 3-month solution (Jaccard = 0.864), whereas the 2-month setting shows lower concordance (Jaccard = 0.640). Relative risks are similar across candidate windows (RR: 2.49–2.66), although LLR values increase slightly with longer windows. Because the observed epidemic peak extends from November 2023 to January 2024 and pediatric MPP shows a clear autumn–winter seasonal pattern, the 3-month window is retained as the main specification, as it matched the main seasonal epidemic phase while maintaining acceptable cluster stability.

The seasonal spatiotemporal clustering analysis covers 46 subdistricts from June 2023 to May 2024, as shown in Table 3. The results show that pediatric MPP incidence rate significantly clusters in the autumn and winter seasons (November 2023—January 2024), accounting for 42.6% of total cases. (In Table 3, RR is the relative risk and LLR is the loglikelihood ratio).

**Table 3:** Spatiotemporal scanning results of seasonal pediatric MPP incidence within the whole study area from June 2023 to May 2024

Location IDs	Time frame	Number of cases	RR	LLR	*p*-value
All	Nov. 1 st, 2023 to Jan. 31 st, 2024	999	2.21	169.2104	0.001

**Fig. 5:**
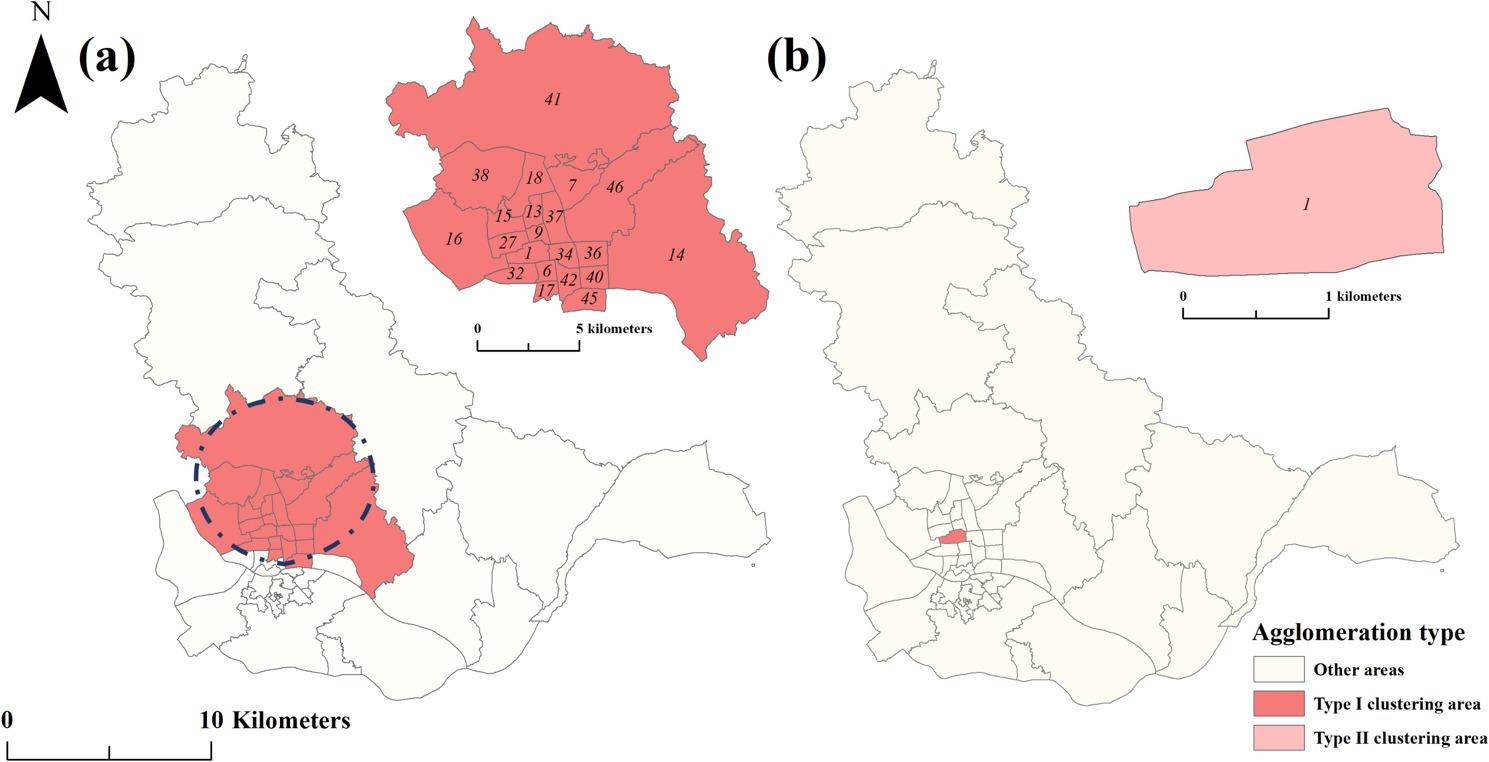
Type I cluster area (**a**) and Type II cluster area (**b**) identified by SaTScan analysis

Table 4 delineates spatiotemporal scan statistics of pediatric MPP incidence across Fuzhou's five administrative districts (June 2023 – May 2024), identifying statistically significant space–time clusters through Kulldorff's scan method. The primary cluster (November 1, 2023 – January 31, 2024) encompasses subdistricts 7, 18, 37, and 46, etc. (circular radius = 10.25 km), capturing 637 cases with elevated relative risk (RR = 2.63, LLR = 183.62, *p* < 0.0001). This robust cluster demonstrates intense winter transmission, geographically covering all subdistricts of Gulou District, southern Jin'an District, and northern Taijiang District as illustrated in Fig. 5a. A secondary cluster (March 1 – April 30, 2024) exhibits explosive risk amplification (RR = 6.78, LLR = 15.89, *p* < 0.0001) within Subdistrict 1, representing focal hyperendemicity despite lower case count (*n* = 15), with spatial distribution detailed in Fig. 5b.

**Table 4:** Spatiotemporal scanning results of pediatric MPP incidence from June 2023 to May 2024

Type	Scanning area	ID	Span (km)	Time frame	Number of cases	RR	LLR	*p*-value
Ⅰ	All	7, 18, 37, 46, 13, 9, 36, 34, 15, 41, 1, 38, 27, 6, 40, 42, 32, 17, 45, 16, 14	10.25	Nov. 1 st, 2023 to Jan. 31 st, 2024	637	2.63	183.62	< 0.00001
Ⅱ		1	0	Mar. 1 st, 2024 to Apr. 30 st, 2024	15	6.78	15.89	< 0.0001
Ⅰ	Jinan	46,7	2.14	Nov. 1 st, 2023 to Jan. 31 st, 2024	131	3.04	52.99	< 0.00001
Ⅱ		14,36	4.18	Dec. 1 st, 2023 to Feb. 29 st, 2024	112	1.53	8.34	0.0066
Ⅰ	Gulou	1	0	Nov. 1 st, 2023 to Mar. 31 st, 2024	41	12.38	64.00	< 0.00001
Ⅱ		38,13,15,18	2.92	Nov. 1 st, 2023 to Mar. 31 st, 2024	120	2.37	28.84	< 0.00001
Ⅰ	Cangshan	21	0	Nov. 1 st, 2023 to Jan. 31 st, 2024	92	3.12	38.63	< 0.00001
Ⅱ		3,10,11,23,39	2.33	Oct. 1 st, 2023 to Dec. 31 st, 2023	48	2.63	15.93	< 0.00001
Ⅰ	Taijiang	5,6,17,42,44,45	2.39	Oct. 1 st, 2023 to Dec. 31 st, 2023	50	2.23	10.53	0.0018
Ⅱ		2,45	2.68	Feb. 1 st, 2024 to Feb. 29th, 2024	10	2.07	2.039	0.998
Ⅰ	Mawei	26	0	Nov. 1 st, 2023 to Jan. 31 st, 2024	60	3.13	22.69	< 0.00001
Ⅱ		26	0	Mar. 1 st, 2023 to Mar. 31 st, 2024	16	2.05	3.11	0.395

**Fig. 6:**
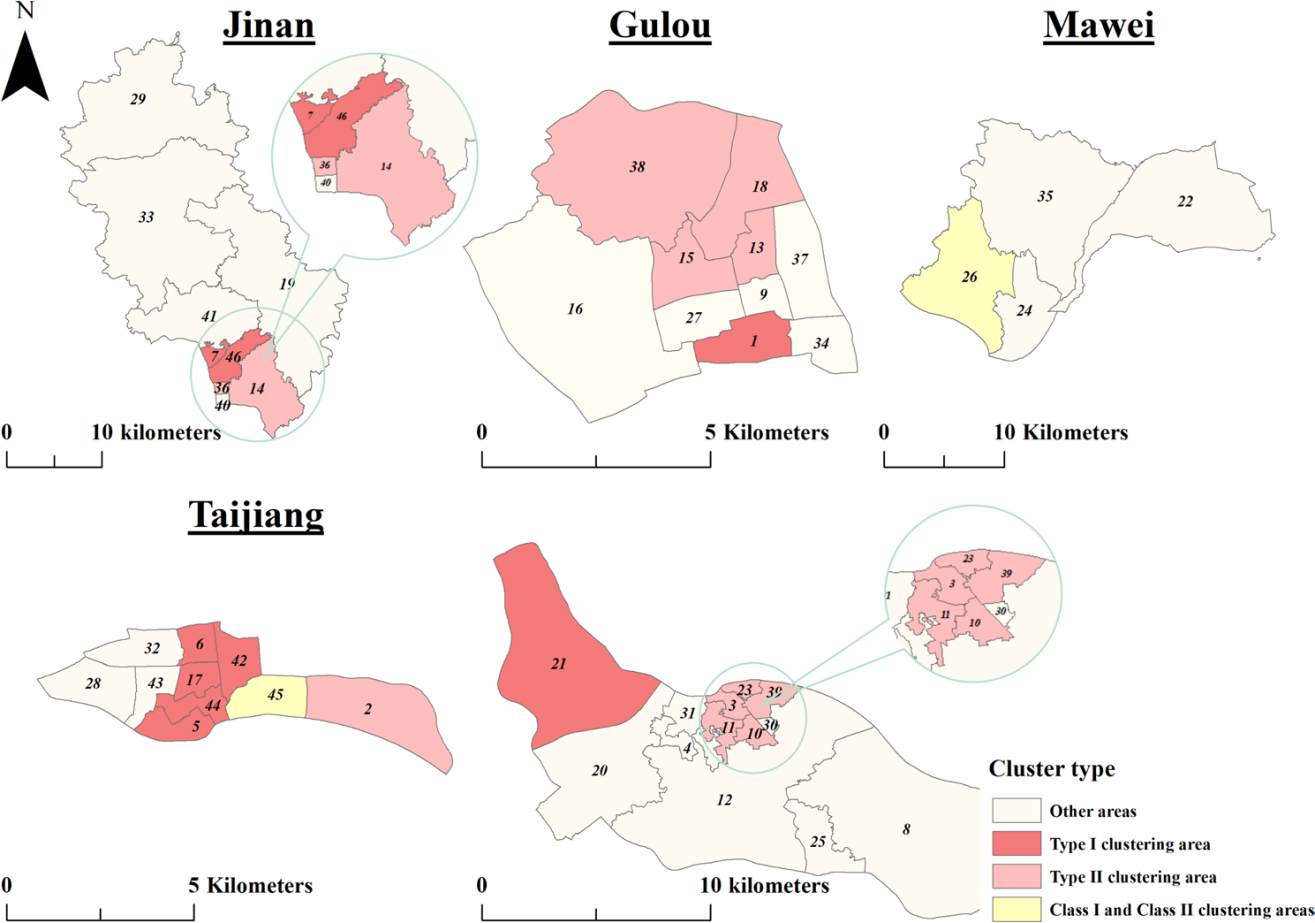
Spatiotemporal scanning results of pediatric MPP incidence in each subdistrict of the study area from June 2023 to May 2024

Substantial heterogeneity in pediatric MPP epidemiology is evidenced by differential Relative Risks (RR), Log–Likelihood Ratios (LLR), and statistical significance across spatiotemporal domains (Table 4, Fig. 6). Jin'an District manifests elevated risk (RR = 3.04, LLR = 52.994, *p* < 0.0001) during November 1, 2023 – January 31, 2024, with southern subdistricts accounting for 78.4% of cases. Gulou District demonstrates extreme risk amplification (RR = 12.3, LLR = 63.998, *p* < 0.0001) from November 1, 2023 to March 31, 2024, with Subdistrict 1 alone constituting 61.3% of district cases. Cangshan District's northern clusters (RR = 3.12, LLR = 38.634, *p* < 0.0001) during November 2023 – January 2024 show significant spatial autocorrelation. Taijiang District exhibits phased transmission patterns: peak risk during October–December 2023 (RR = 5.22, LLR = 10.525, *p* = 0.0018) in central–eastern zones, followed by non–significant decline in February 2024 (RR = 2.07, *p* = 0.998). Mawei District displays transient epidemicity risk between November 2023 and January 2024 (RR = 3.13, LLR = 22.687, *p* < 0.0001) concentrated in Subdistrict 26, diminishing by March 2024 (RR = 1.5, *p* = 0.395). Epidemiological synthesis identifies Jin'an, Gulou, and Cangshan as persistent high–risk zones, contrasting with Taijiang and Mawei's oscillating risk profiles.

### Urban environmental correlates of pediatric MPP onset and severe progression

#### Performance comparison of machine learning models for pediatric MPP incidence analysis

Within the Class I space–time clusters identified earlier—which are located in the hospital’s primary urban catchment area and characterized by broadly comparable socioeconomic status (SES), i.e., minimal between-area SES heterogeneity—we quantified built-environment exposures using buffer analysis. Then, these exposures were assessed for their associations with pediatric MPP onset and progression to severe disease within a comparative, interpretable machine-learning framework. Four machine learning models (i.e., Naïve Bayes, L1-regularized logistic regression, Random Forest, and XGBoost) were benchmarked under stratified five-fold cross-validation, with prespecified inner tuning, class-balanced learning objectives where applicable, and Platt (sigmoid) calibration. Molde performance was evaluated using ROC-AUC, PR-AUC, Brier score; calibration (intercept/slope and plots); operating characteristics at a prespecified 80% sensitivity; and net benefit from decision curve analysis (DCA). The top-performing model was refit on the full dataset and interpreted using TreeSHAP with mean absolute SHAP values, dependence plots, and violin plots. Based on these results, XGBoost was selected as the final model (see Table 5 and Figs. S3-S8). This design—by restricting analyses to SES-homogeneous high-incidence clusters—helps mitigate confounding by socioeconomic status while preserving comparability of environmental effects.

**Table 5:** Comparison of predictive performance of four machine learning models

Model Indicators	XGB	RF	L1_Logit	NB
ROC_AUC	0.784/0.734	0.775/0.706	0.688/0.707	0.524/0.584
PR_AUC	0.909/0.63	0.902/0.593	0.867/0.616	0.794/0.452
Brier	0.132/0.2	0.133/0.209	0.156/0.207	0.172/0.233
Calib_slope	0.184/1.119	1.164/1.117	1.224/1.104	0.781/0.974
Calib_intercept	−0.181/0.045	−0.162/0.034	−0.258/0.034	0.274/−0.017
Thr@Sens80%	0.788/0.32	0.798/0.303	0.73/0.31	0.77/0.341
Sensitivity	0.799/0.798	0.799/0.802	0.801/0.798	0.805/0.798
Specificity	0.661/0.519	0.661/0.445	0.489/0.506	0.194/0.278
PPV	0.893/0.514	0.893/0.48	0.847/0.508	0.78/0.413
NPV	0.482/0.802	0.482/0.779	0.409/0.798	0.22/0.684

Note: The values on the left side of the table are the prediction results of the model for MPP onset, and the values on the right side are the prediction results for the progression of MPP to severe disease.

#### Built-environment correlates of pediatric MPP onset and severe progression

**Fig. 7:**
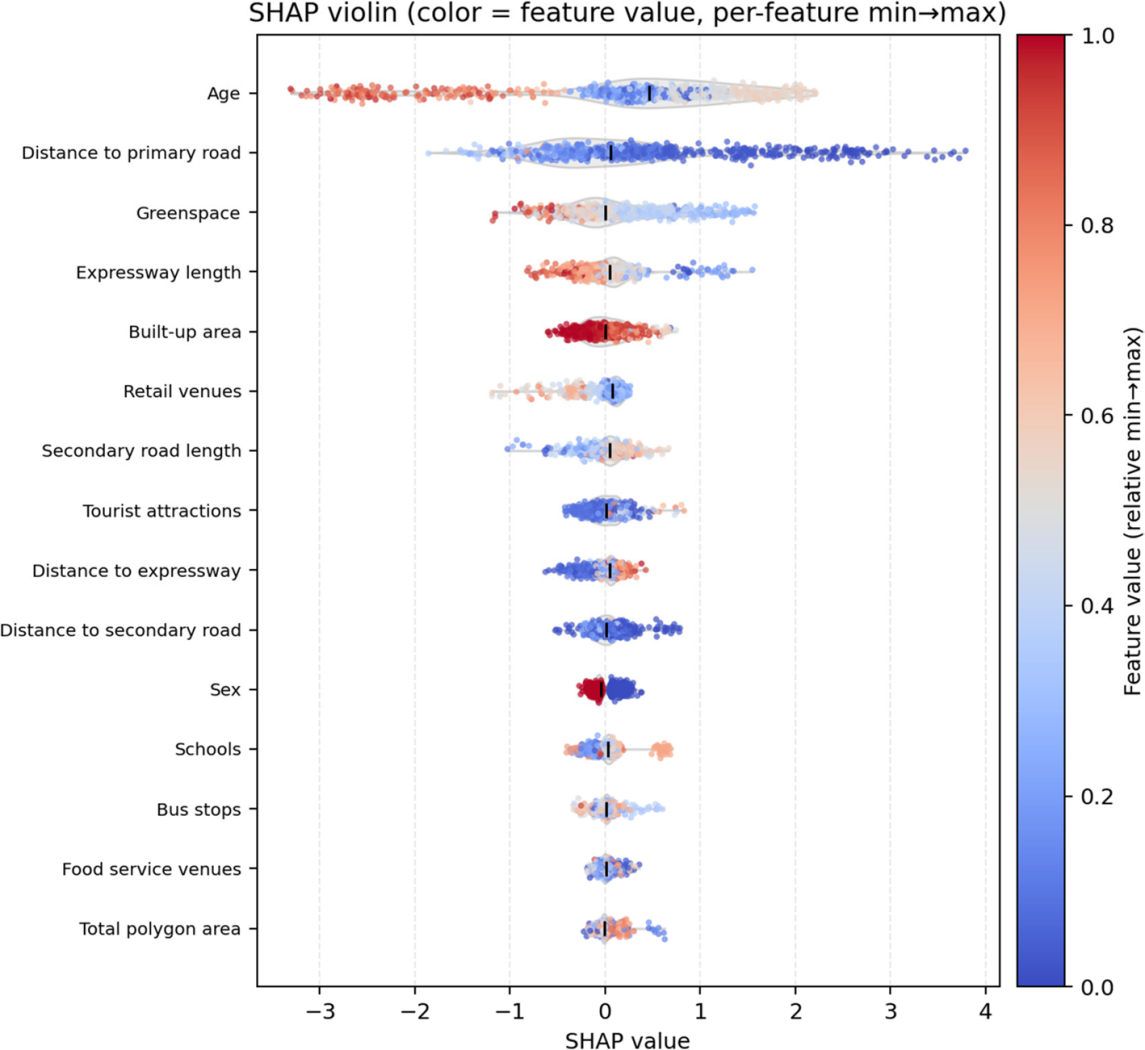
SHAP analysis of associations between urban environmental factors and pediatric MPP onset

In the well-built XGBoost model based on all the MPP samples, the SHAP summary presented in Fig. 7 and Fig. S9 identifies age as the dominant predictor determing pediatric MPP onset, with increasing age yielding negative contributions on the log-odds scale (i.e., lower predicted risk among older children). Accessibility variables display coherent gradients: shorter distances to primary roads (and weakly to secondary roads and expressways) are associated with positive SHAP values. Urban-intensity metrics, including expressway length, built-up area, and retail-venue density, show moderate positive contributions. In contrast, greenspace shows a consistently negative contribution. The remaining features (i.e., sex, schools, bus stops, food-service venues, and total polygon area) have SHAP values near zero and are therefore of minor importance. These patterns characterize the model’s decision surface and represent associations, not causal effects.

**Fig. 8:**
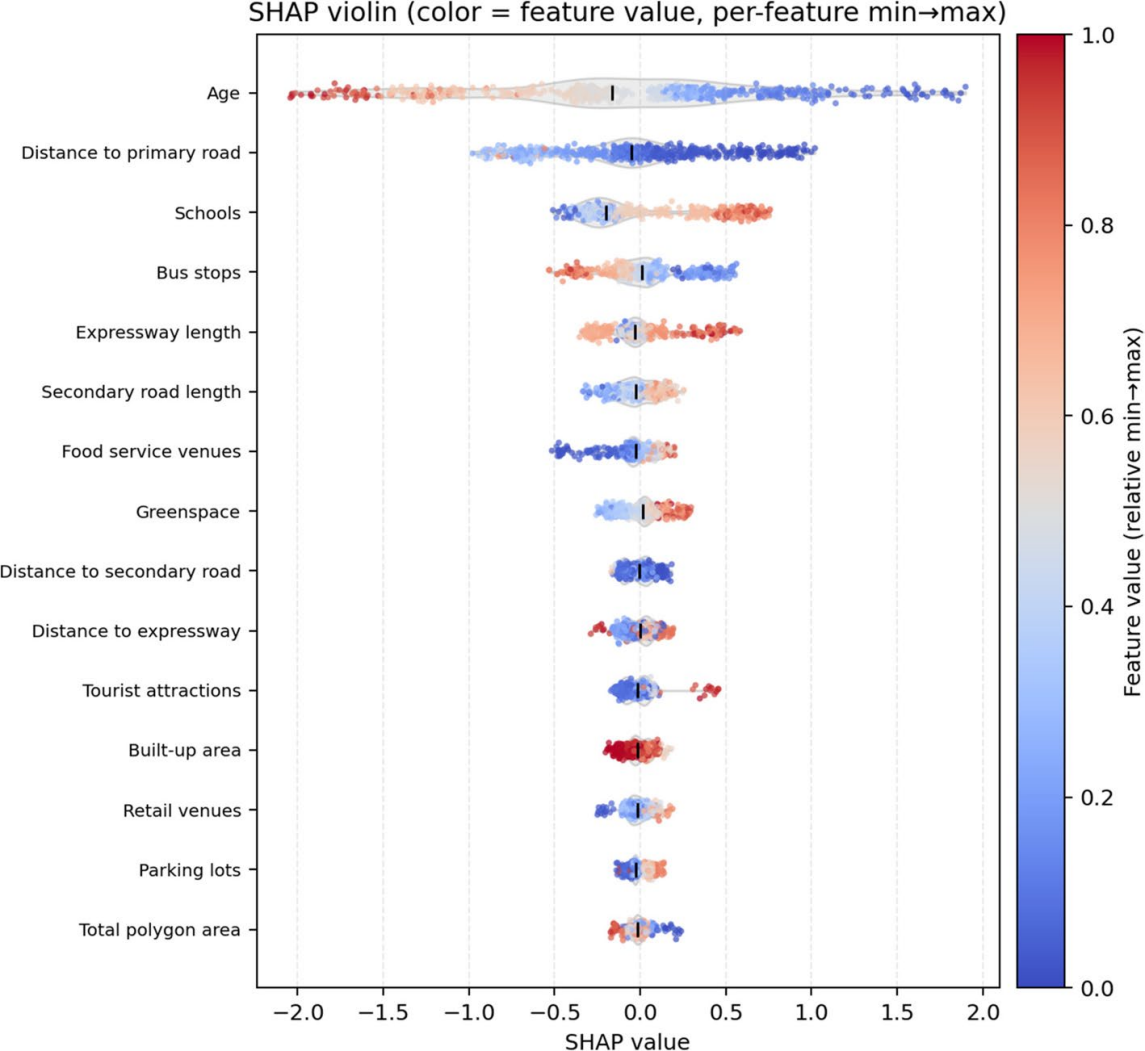
SHAP analysis of associations between urban environmental factors and pediatric MPP progression to severe pneumonia

In the model of severe disease progression (see Fig. 8 and Fig. S10), SHAP analysis indicates that age is the most influential predictor and exhibits a clear negative gradient—higher ages cluster at negative SHAP values (protective), whereas younger ages are positive. Distance to primary road ranks second and shows a strongly monotonic: closer proximity yields positive SHAP values and greater distances are negative. Number of schools ranks third, with more schools showing small-to-moderate positive contributions and lower school counts near zero to slightly negative. Food-service venue density contributes little, remaining near zero with a mild negative tendency at higher densities. Greenspace becomes more negative at higher levels, and total polygon area likewise trends negative as area increases. Other features (e.g., bus stops, expressway length, secondary road length, and distances to secondary roads/expressways) show weak, near-zero effects. Collectively, predicted progression risk is most strongly associated with younger age and near-road exposure, with school density adding a smaller but consistent positive effect; remaining attributes make only minor adjustments.

#### SHAP dependence patterns of key predictors for pediatric MPP onset and severe progression

**Fig. 9:**
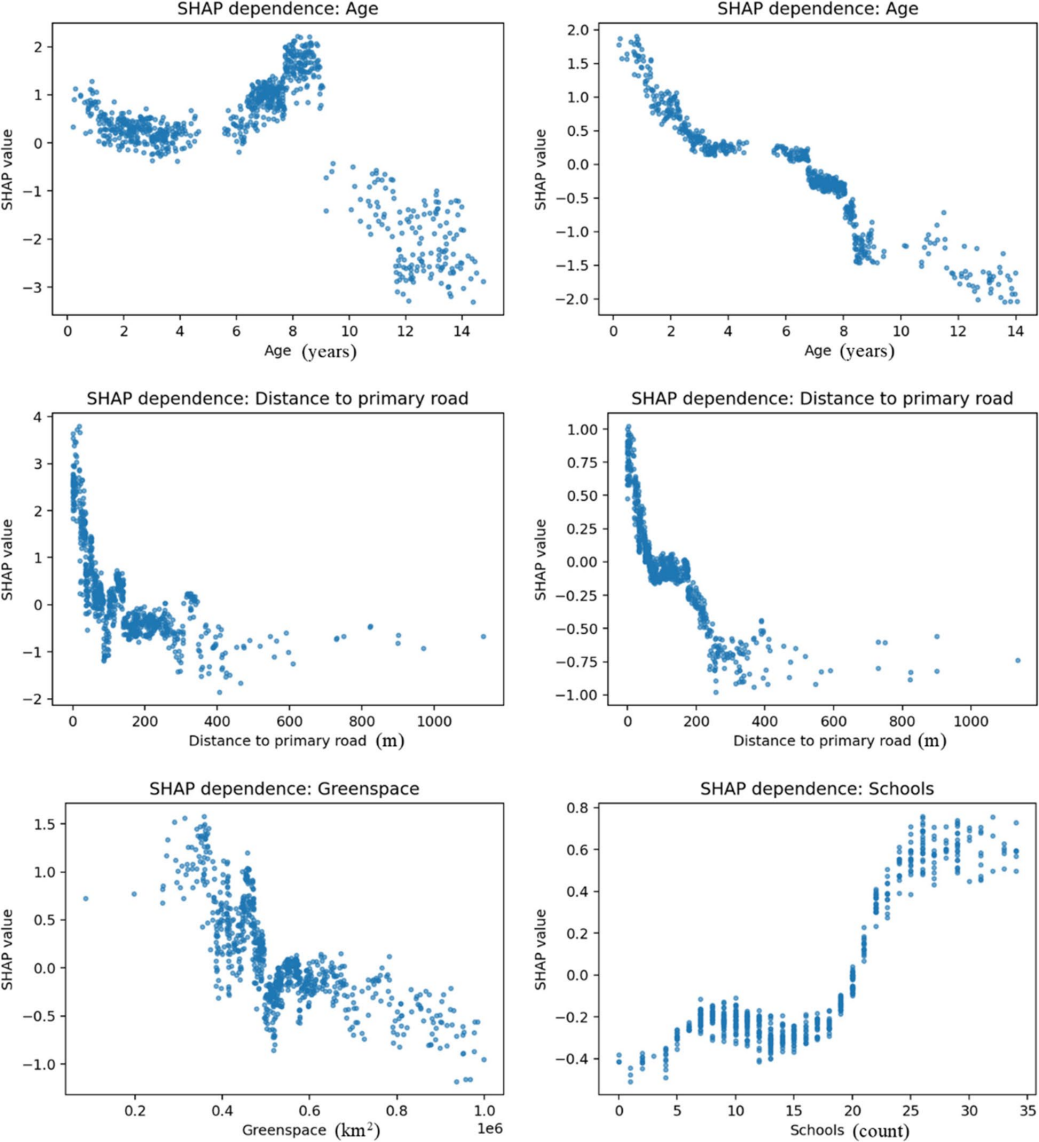
SHAP dependence plots for key predictors of two MPP stages: onset (left) vs. progression to severe disease (right)

**Fig. 10:**
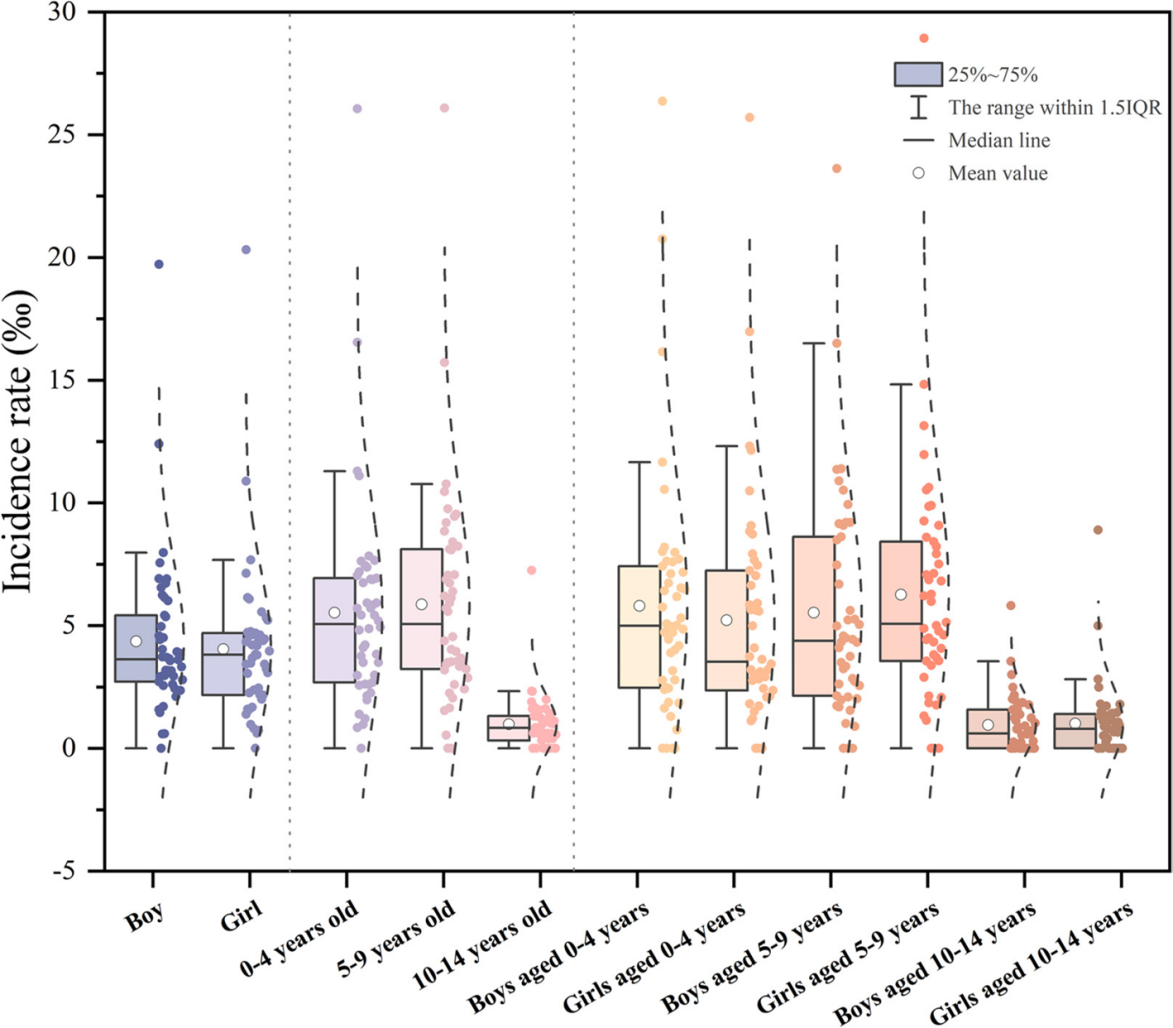
Population characteristics of pediatric MPP onset among subdistricts in the study area from June 2023 to May 2024

Fig. 9 presents SHAP dependence plots for modelling the MPP incidence onset (left) and severe-progression (right). In the incidence onset model, age shows a pronounced non-linear pattern. SHAP values are predominantly positive at 5–9 years and become strongly negative above ≈10 years. Distance to primary road displays a steep inverse gradient, with SHAP values turning from strongly positive at very short distances to negative and eventually plateauing as distance increases. Greenspace shows an approximately monotonic declines, with greater greenness associated with increasingly negative contributions. In the severe progression model, age exhibits a clear negative gradient (younger ages positive, older ages negative); distance to primary road shows the same inverse relationship as in the incidence onset model but with a smaller amplitude; and school counts demonstrate an approximately monotonic increasing, sigmoid-like association with SHAP values. These patterns describe model behavior (associations) rather than causal effects. Because age emerged as the leading predictor within Class I clusters, we profiled age- and sex-specific patterns across the full study area. Fig. 10 further shows subdistrict-level MPP onset distributions stratified by sex and age. Boys have slightly higher medians and means than girls, as well as greater dispersion, indicating greater between-subdistrict heterogeneity. MPP onset peaks in the 5–9 years group, is lower in the 0–4 years group, and is lowest in the 10–14 years group, with the latter showing the most compact distributions. Within-age comparisons indicate the largest sex differences in the 5–9 and 10–14 years strata (boys > girls), with minimal differences in 0–4 years. Subdistricts 1 and 46 recur as high-incidence outliers across several strata, particularly among 5–9-year boys and girls.

## Discussion and implications

This study demonstrates pronounced seasonal variation in pediatric MPP incidence across 46 urban subdistricts in Fuzhou, with the nadir in summer and epidemic peaks during autumn–winter transition (weekly incidence rate = 35.5‰). Spring exhibits intermediate transmission intensity, 1.4–fold higher than summer. This seasonal pattern aligns with global MPP epidemiology [6], reflecting synergistic environmental and behavioral drivers. The winter peak period is associated with factors such as cold weather, fluctuations in air pollution levels, indoor aggregation patterns of children, and the humid and cold climate of Fuzhou that may prolong the airborne survival time of pathogens [30]. During the cold season, air pollutant concentrations are typically elevated, further increasing the risk of infection among children [31, 32]. The secondary peaks in autumn coincide with immune-challenging diurnal temperature shifts and school-mediated population aggregation, amplifying transmission. Spring vulnerability arises from unstable thermal conditions and pollen-induced mucosal inflammation. After the winter holiday, children returning to school may carry pathogens in the incubation period, potentially triggering localized outbreaks. Summer reductions reflect outdoor activity patterns, school closures, and strong ultraviolet radiation, collectively limiting pathogen survival and contact density.

In terms of spatiotemporal clusters, this study reveals that although the incidence of pediatric MPP varies significantly across seasons, high–incidence areas are predominantly concentrated in the central urban regions, such as southern of Jinan District, Gulou District, and northern of Taijiang District consistently forming a stable high–risk cluster. The global autocorrelation results indicate that pediatric MPP incidence was more strongly structured by adjacent among neighboring subdistricts than by smooth distance-decay alone. At the same time, the modest magnitude of the global Moran’s I statistic does not contradict the presence of meaningful local hotspots, and may in part reflect the relatively small number of spatial units (i.e., 46 subdistricts). Against this background, the central urban areas were identified as a type I cluster during both autumn and winter and exhibits sustained high aggregation throughout the year, indicating that children residing in this region face a persistently elevated risk of MPP infection. Several factors may underline this phenomenon, including high population density, dense road networks contributing to poor air quality, opaque urban structures, and complex neighborhood environments. In contrast, peripheral subregions display low–risk clusters. These findings align with previous research, which suggests that areas characterized by high population density, limited green spaces, and poor air quality are more susceptible to respiratory diseases [33–35]. Furthermore, the spatial extent of high incidence during autumn is greater than in other seasons. This can be attributed to the sharp decline in temperatures and the significant diurnal temperature fluctuations (daily variations reaching 8–10℃) during autumn in Fuzhou, as well as to spatial heterogeneity in microclimatic conditions–such as humidity and temperature gradients between mountainous and urban areas–that unevenly influence pathogen transmission. Additionally, the beginning of the school term after summer break, when children return from dispersed living arrangements (e.g., visiting hometowns or traveling), likely facilitates the synchronous introduction of pathogens into multiple areas, leading to multi–focal outbreaks. It is noteworthy that Subdistricts 1 and 46 are consistently identified as high–outbreak zones, with incidence rates substantially exceeding those in other areas. This may be related to the high concentration of daycare centers and preschools and elevated building density in these subdistricts, which collectively promote frequent close–contact interactions among children in both indoor and outdoor settings [36, 37]. Within the five-district service area, MPP incidence shows a moderate negative rank correlation with distance (Spearman’s ρ = − 0.36, *p* < 0.001), yet the functional form is non-monotonic trend, with a clear peak at approximately 5–6 km (city center) and relatively flat values at shorter and longer distances. By contrast, MPP case counts exhibit no monotonic association with distance (ρ = − 0.067, *p* = 0.746). These descriptive checks do not support the monotonic distance-decay expected under strong systematic under-ascertainment. A similar strengthening pattern was observed for near-road exposure. In the incidence onset model, shorter distance to primary roads shows stable positive SHAP contributions, consistent with traffic-related air pollution in roadside microenvironments. Prior studies link residential roadway context to airway inflammation in children (elevated exhaled NOx) [38–40], and near-road monitoring demonstrates steep pollutant gradients within a few hundred meters of major roads. In the severe-progression model, distance to primary road ranks second and shows a steeper inverse dependence: very short distances is strongly positive, SHAP values cross zero around 100–150 m and then plateau below zero as distance increases. This pattern is consistent with field evidence that concentrations of near-road pollutants such as NO_x_, ultrafine particles, and black carbon decline rapidly within the first 100–150 m from major roads and continue to attenuate over the following few hundred meters, while restricted ventilation in street-canyon environments can further intensify pollutant accumulation close to roads [41–47]. In this context, near-road exposure may be relevant to both disease occurrence and progression by increasing exposure to airway irritants and pro-inflammatory pollutants, which could heighten respiratory vulnerability, amplify airway inflammation, and contribute to more severe clinical progression once infection has occurred [15, 16]. Under this interpretation, near-road exposure appears to be relevant not only to MPP occurrence, but also more strongly to progression once infection has occurred, plausibly through sustained airway irritation and inflammatory burden. The remaining built-environment variables show more stage-specific patterns. In the incidence onset model, greenspace is negatively associated with risk, in line with reviews and school-based measurements showing lower indoor TRAP where greenness is higher [48, 49]. The greenspace metric was derived from the validated 1 m UGS-1 m dataset, which improves neighborhood-level spatial detail. However, it remained an aggregate exposure measure and did not capture greenspace type, accessibility, quality, vegetation structure, maintenance, or actual use. Therefore, the observed negative association should not be interpreted as a specific protective effect of greenspace itself. In contrast, in the severe-progression model, school density ranks third and retains an approximately monotonic positive dependence, indicating that neighborhoods with more schools are associated with higher predicted progression risk. However, because we lacked data on school attendance, extracurricular activities, commuting behavior, or direct contact networks, this variable should be interpreted cautiously as a neighborhood-level proxy for child-oriented activity environments rather than a direct measure of interpersonal mixing. Collectively, the modelling anaysis suggests a progression from broader incidence-related urban correlates toward a more concentrated severe-risk profile, characterized by younger age, very near-road residence, and a child-oriented neighborhood context. We therefore interpret these findings as model-based association patterns that help distinguish factors linked to MPP occurrence from those more strongly associated with severe progression, rather than as direct evidence of causal biological effects.

It is important to note that the SHAP analysis results reflect conditional contributions within the learned covariance structure and should not be interpreted as causal effects. As such, SHAP values in this study are best understood as model-based explanations rather than estimates of biological effects. Because several built-environment variables remain correlated to some extent even after feature screening, attribution may be shared or redistributed across related predictors, and thus, SHAP rankings should not be interpreted as isolated exposure effects. In addition, SHAP dependence plots partly reflect interaction structure or shared covariance among predictors rather than pure main effects. As a result, we treat these SHAP patterns as conditional association signals useful for hypothesis generation, not as direct evidence of causal or biological mechanisms.

Taken together across both models based on all incidence cases and severe progression of pediatric MPP, near-road exposure repeatedly shows positive associations, whereas greenspace is consistently negative. These patterns align with existing evidence on traffic-related air pollution and street-canyon dispersion, which may jointly shape both infection acquisition and disease progression.

## Study limitations

This study has several limitations. Our exposure assessment was based on residential location and neighborhood-scale spatial indicators, which describe the surrounding urban context but cannot fully reflect children’s daily mobility, school attendance, commuting routes, or time spent in different environments. In addition, some environmental measures remained broad indicators. For example, the greenspace indicator did not distinguish type, accessibility, quality, or actual use, and school density should be understood as a neighborhood-level proxy rather than a direct measure of interpersonal contact. Finally, although we combined spatial statistics with interpretable machine learning, this study was still observational, and the SHAP patterns should be interpreted as model-based associations rather than causal effects. Despite these limitations, the study still provides a useful neighborhood-scale picture of pediatric MPP spatiotemporal patterns and their urban environmental correlates in the main urban area of Fuzhou, China.

## Conclusions

This study integrates spatial statistics with interpretable machine learning to characterize subdistrict-level pediatric MPP distribution in Fuzhou, China. We observed pronounced seasonality with an autumn–winter peak, consistent with local climate, indoor crowding, and school-term rhythms. Persistent clustering in central districts was associated with high population density, dense transport networks, limited green space, and elevated traffic pollutant loads. Boys and children aged 5–9 years showed relatively higher incidence. Machine learning identified age as the most informative predictor, showing a non-monotonic association with incidence onset and a negative gradient with severe progression of pediatric MPP. Distance to primary road ranked second, particularly in the severe progression stage, showing a steep inverse gradient: strongly positive at very short distances, becoming near-zero at 100–150 m, then tapering to a negative plateau. School density ranked third, with an approximately monotonic positive association. Other accessibility proxies contributed weak, near-zero signals. Greenspace showed a consistent negative association, while service-industry densities showed a saturation-like decrease, with limited incremental value once mixing and proximity were captured. Based on these findings, policy implications include seasonally tailored air-quality management especially in autumn and winter, strengthened school-based health monitoring during epidemic periods, and targeted urban interventions in high-prevalence clusters—particularly near-road exposure mitigation (ventilation, filtration, buffers, setbacks, traffic management) and context-specific greening. Future work should further prioritize longitudinal exposure assessment, interaction-aware validation (e.g., age  × distance, age  × schools), and policy evaluation.

## Supplementary Information

Supplementary Material 1.

## Data Availability

Availability of data and materials： The datasets generated and/or analyzed during the current study are not publicly available because they contain de-identified pediatric clinical and geospatially derived data and are subject to privacy, ethical, and institutional restrictions. The data may be available from the corresponding author on reasonable request and with permission from Fujian Children’s Hospital.

## Abbreviations

CAP: Community-acquired pneumonia
MPP: Mycoplasma pneumoniae pneumonia
POI: Points of interest
SHAP: Shapley Additive explanations
RR: Relative risk
LLR: Log-likelihood ratio
ROC-AUC: Area under the receiver operating characteristic curve
PR-AUC: Area under the precision–recall curve
DCA: Decision curve analysis
SES: Socioeconomic status
