# Supplementary material for: Urban environmental and climatic correlates of pediatric respiratory infection: microscale spatiotemporal evidence from Fuzhou, China
Source: BMC Pediatr. 2026 May 30;26:704. doi: 10.1186/s12887-026-06993-2 (PMC13435392; doi:10.1186/s12887-026-06993-2)
Supplement: Supplementary file 1 [file 12887_2026_6993_MOESM1_ESM.docx]

<Appendix A Supplementary Material>

**Urban environmental and climatic correlates of pediatric respiratory infection: Microscale spatiotemporal evidence from Fuzhou, China**

Yangbin Dong ^a,#^, Yanying Rao ^b,#^, Jie Zhang ^a^, Qiuyu Tang ^c,**^, Zhanyong Wang ^a,*^

^a^ College of Transportation and Civil Engineering, Fujian Agriculture and Forestry University, Fuzhou 350108, China

^b^ Department of Radiology, Fujian Children's Hospital (Fujian Branch of Shanghai Children's Medical Center), College of Clinical Medicine for Obstetrics & Gynecology and Pediatrics, Fujian Medical University, Fuzhou 350014, Fujian, China

^c^ Pediatric Intensive Care Unit‌, Fujian Children's Hospital (Fujian Branch of Shanghai Children's Medical Center), College of Clinical Medicine for Obstetrics & Gynecology and Pediatrics, Fujian Medical University, Fuzhou 350014, Fujian, China

\# These authors contributed equally to this work.

*Corresponding authors: tell.rain@163.com (Q. Tang); wangzy1026@fafu.edu.cn (Z. Wang).

**Fig.S1** Distance to hospital vs. number of cases

**Fig.S2** Distance to hospital vs. incidence rate (‰)

**Table S1** Cluster membership under alternative maximum temporal windows in the space–time scan analysis

| **Time window** | **Type** | **Scanning area** | **Subdistrict IDs** | **Span (km)** | **Time frame** | **Number of cases** | **RR** | **LLR** | **p-value** |
| --- | --- | --- | --- | --- | --- | --- | --- | --- | --- |
| 3 months | Ⅰ | All | 7, 18, 37, 46, 13, 9, 36, 34, 15, 41, 1, 38, 27, 6, 40, 42, 32, 17, 45, 16, 14 | 10.25 | 2023/11/1 to 2024/1/31 | 637 | 2.63 | 183.62 | < 0.00001 |
|  | Ⅱ |  | 1 | 0 | 2024/3/1 to 2024/4/30 | 15 | 6.78 | 15.89 | <0.0001 |
| 1 month | Ⅰ | All | 46, 7, 37, 36, 18, 13, 34, 14, 9, 40, 42, 1, 6, 15, 41, 45, 27, 2,17, 38 | 11.21 | 2023/12/1 to 2023/12/31 | 224 | 2.65 | 74.52 | < 0.00001 |
|  | Ⅱ |  | 7, 18, 37, 46 | 4.12 | 2023/10/1 to 2023/10/31 | 50 | 2.60 | 16.81 | <0.0001 |
| 2 months | Ⅰ | All | 29, 33, 19, 41, 38, 18, 7, 46, 13, 15, 37, 16, 9, 27, 1, 34, 36, 32,6, 21 | 10.25 | 2023/11/1 to 2023/12/31 | 426 | 2.66 | 183.62 | < 0.00001 |
|  | Ⅱ |  | 1 | 0 | 2024/2/1 to 2024/3/31 | 22 | 10.14 | 31.05 | <0.0001 |
| 4 months | Ⅰ | All | 46, 7, 37, 36, 18, 13, 34, 14, 9, 40, 42, 1, 6, 15, 41, 45, 27, 2,17, 38 | 11.21 | 2023/10/1 to 2024/1/31 | 742 | 2.58 | 198.34 | < 0.00001 |
|  | Ⅱ |  | 1 | 0 | 2024/3/1 to 2024/4/30 | 15 | 6.78 | 15.89 | <0.0001 |
| 5 months | Ⅰ | All | 46, 7, 37, 36, 18, 13, 34, 14, 9, 40, 42, 1, 6, 15, 41, 45, 27, 2,17, 38 | 11.21 | 2023/10/1 to 2024/2/29 | 637 | 2.49 | 203.86 | < 0.00001 |
|  | Ⅱ |  | 3, 23, 11, 5, 44, 39, 10 | 3.13 | 2023/10/1 to 2023/12/31 | 15 | 1.73 | 8.45 | 0.117 |

**Table S2.** Sensitivity of space–time scan results to maximum temporal window setting

| **Max temporal window** | **Primary-cluster time frame** | **Primary-cluster RR** | **Primary-cluster LLR** | **No. of subdistricts** | **Jaccard vs. 3-month** |
| --- | --- | --- | --- | --- | --- |
| 1 month | 2023/12/1–2023/12/31 | 2.65 | 74.52 | 20 | 0.864 |
| 2 months | 2023/11/1–2023/12/31 | 2.66 | 183.62 | 20 | 0.640 |
| 3 months | 2023/11/1–2024/1/31 | 2.63 | 183.62 | 21 | 1.000 |
| 4 months | 2023/10/1–2024/1/31 | 2.58 | 198.34 | 20 | 0.864 |
| 5 months | 2023/10/1–2024/2/29 | 2.49 | 203.86 | 20 | 0.864 |

**Note:** RR, relative risk; LLR, log-likelihood ratio. Jaccard similarity was calculated using the overlap in primary-cluster membership relative to the 3-month setting.

**Table S3.** Hyperparameter tuning and calibration scheme for the machine-learning models

| **Model** | **Preprocessing** | **Handling of class imbalance** | **Hyperparameters tuned** | **Candidate settings considered** | **Tuning strategy** | **Selection metric** | **Probability calibration** |
| --- | --- | --- | --- | --- | --- | --- | --- |
| Naïve Bayes | Median imputation | No additional weighting applied | None | Not applicable | Default specification | Not applicable | Platt scaling on training data |
| L1-Logit | Median imputation; z-standardization | Class-balanced weighting | Regularization strength | Log-scaled candidate values | Prespecified tuning with 3-fold stratified inner CV | Mean ROC-AUC | Platt scaling on training data |
| Random Forest | Median imputation | Class-balanced weighting | Number of trees, minimum leaf size, split features | Prespecified candidate values | Prespecified tuning with 3-fold stratified inner CV | Mean ROC-AUC | Platt scaling on training data |
| XGBoost | Median imputation | Positive-class weighting based on training-set class ratio | Boosting rounds, tree depth, learning rate, row/column subsampling, minimum child weight, L2 regularization, split-gain threshold | Prespecified candidate values | Prespecified tuning with 3-fold stratified inner CV | Mean ROC-AUC | Platt scaling on training data |

**note:** All models were evaluated using stratified five-fold outer cross-validation. Hyperparameter tuning, where applicable, was conducted only within the training folds. Final model performance was summarized from out-of-fold predictions. The evaluation framework included ROC-AUC, PR-AUC, calibration slope and intercept, operating characteristics at 80% sensitivity, and decision curve analysis.

**Fig.S3** ROC curves (K-fold out-of-fold) comparing models for pediatric MPP incidence

**Fig.S4** Calibration curves (K-fold CV) for MPP risk models

**Fig.S5** Decision curve analysis (K-fold CV) for MPP risk models

**Fig.S6** ROC curves (K-fold out-of-fold) comparing models for progression from MPP to severe pneumonia

**Fig.S7** Calibration curves (K-fold CV) for models predicting progression from MPP to severe pneumonia

**Fig.S8** Decision curve analysis (K-fold CV) for models predicting progression from MPP to severe pneumonia

**Fig.S9** Top 15 features for pediatric MPP incidence (ranked by mean |SHAP|, XGBoost)

**Fig.S10** Top 15 features for progression from MPP to severe pneumonia (ranked by mean |SHAP|, XGBoost)
